# PREDICTION OF INDEPENDENT AMBULATION IN SUBACUTE STROKE PATIENTS: A RETROSPECTIVE OBSERVATIONAL STUDY USING LOWER LIMB MOTOR AND BALANCE ASSESSMENTS

**DOI:** 10.2340/jrm.v57.44054

**Published:** 2025-08-05

**Authors:** Jungwoo SHIM

**Affiliations:** Department of Rehabilitation Medicine, Chungnam National University Sejong Hospital, Bodeum, Sejong-si, Republic of Korea

**Keywords:** ambulation, balance, Berg balance scale, Fugl-Meyer assessment, gait, rehabilitation, stroke

## Abstract

**Objective:**

To investigate whether motor and balance assessments predict independent ambulation in patients with subacute stroke.

**Design:**

Retrospective observational study.

**Subjects/Patients:**

109 patients admitted for inpatient rehabilitation within 3 months of stroke onset.

**Methods:**

Independent ambulation was defined as a Functional Ambulation Category score of 4 or higher on discharge. The Fugl-Meyer Assessment for Lower Limb and Berg Balance Scale were administered on admission. Associations with walking independence on both admission and discharge were examined using binary logistic regression, adjusted for age, sex, and Korean Mini-Mental State Examination scores. Discriminative ability was assessed using receiver operating characteristic curve analysis.

**Results:**

On admission, both the Fugl-Meyer Assessment for Lower Limb (*B* = 0.42, *p* < 0.001, odds ratio = 1.52) and the Berg Balance Scale (*B* = 0.31, *p* < 0.001, odds ratio = 1.37) were significantly associated with walking independence. Both assessments also significantly predicted independence in ambulation on discharge. The Berg Balance Scale showed excellent discriminative performance (area under the curve = 0.97 on admission, 0.88 on discharge), and the Fugl-Meyer Assessment also performed well (area under the curve = 0.89 and 0.82).

**Conclusion:**

Admission motor and balance assessments are significant predictors of walking independence in subacute stroke patients and may inform early rehabilitation decisions.

## LAY ABSTRACT

Walking again after a stroke is a key goal in rehabilitation. This study examined whether 2 simple clinical tests, 1 that measures leg movement and another that assesses balance, can help predict whether a patient will eventually walk independently. The researchers reviewed medical record 109 stroke patients and analysed how well the test results matched their walking outcomes. They found that both assessments were useful, with the balance test showing particularly strong accuracy. These findings suggest that early evaluations, even before a person can walk, may help therapists plan effective treatments, set realistic goals, and decide when to begin walking-focused rehabilitation.

Stroke is a major cause of long-term disability, often resulting in significant impairments in mobility and quality of life. Among the various sequelae, the loss of independent ambulation is considered a critical barrier to functional autonomy and community reintegration. Therefore, early prediction of gait recovery is essential for establishing realistic rehabilitation goals and optimizing treatment strategies (1, 2).

The subacute phase, particularly within the first 3 months after stroke onset, is considered a key window for neurological recovery (3). During this period, clinical tools that assess lower limb motor and balance functions play an important role in predicting recovery potential (3, 4). The Fugl-Meyer Assessment for Lower Limb (FMA-L/L) and the Berg Balance Scale (BBS) are widely used and validated instruments for evaluating lower limb motor control and balance, respectively (5, 6). However, their ability to predict independent ambulation, defined as a Functional Ambulation Category (FAC) score of 4 or higher, has not been sufficiently investigated.

This study aimed to evaluate the clinical utility of the FMA-L/L and the BBS in relation to independent ambulation among patients with subacute stroke. Specifically, we investigated both the concurrent association between admission scores and ambulation status on admission, and the predictive value of admission scores for ambulation outcomes on discharge.

The results of this study may contribute to evidence-based rehabilitation planning and clinical decision-making by supporting the use of simple, standardized assessment tools in early stroke recovery.

## METHODS

### Study design and participants

This retrospective observational study was conducted at the Department of Rehabilitation Medicine, Chungnam National University Sejong Hospital. Patients were eligible for inclusion if they had experienced a first-ever ischaemic or haemorrhagic stroke confirmed by brain imaging (computed tomography or magnetic resonance imaging), were within 3 months of stroke onset (subacute phase), and had complete data available for the FMA-L/L, the BBS, and the FAC. Patients were excluded if they had a history of recurrent stroke, had been dependent in walking ability prior to stroke onset, had severe cognitive impairment as indicated by a Korean Mini-Mental State Examination (K-MMSE) score less than 10, or had coexisting neurological or musculoskeletal disorders that could significantly interfere with gait or balance function. A total of 211 patients admitted between July 2020 and April 2023 were initially screened. Of these, 102 were excluded due to incomplete functional assessment data (*n* = 61), recurrent stroke (*n* = 23), pre-stroke ambulatory dependence (*n* = 10), and severe cognitive impairment (K-MMSE < 10; *n* = 8). As a result, 109 patients were included in the final analysis. Cases with missing or incomplete data were excluded using listwise deletion. To minimize selection bias, all eligible patients during the study period were enrolled consecutively. This study was approved by the Institutional Review Board of Chungnam National University Sejong Hospital (IRB No. 2023-05-008), and the requirement for informed consent was waived due to the retrospective nature of the study. The sample size was not determined *a priori* due to the retrospective nature of the study. Instead, all eligible patients who met the inclusion criteria during the study period were consecutively included to minimize selection bias.

### Outcome measure

The primary outcome was independent ambulation on discharge. This was defined as a score of 4 or greater on the FAC, which is a 6-point ordinal scale that classifies walking ability from complete dependence to full independence, with a score of 0 representing non-functional ambulation and a score of 5 indicating independent walking on all surfaces without supervision (7).

### Assessment variables

The main clinical variables were the FMA-L/L and BBS, both of which were measured on admission and again on discharge. The FMA-L/L is a reliable tool used in stroke rehabilitation to assess lower limb motor function, with scores ranging from 0 to 34 points. Higher scores indicate better motor function (8). The BBS assesses balance ability on a 56-point scale, with higher scores indicating greater balance ability (9). It is commonly used in both clinical and research settings to estimate fall risk and mobility potential. In order to account for potential confounding variables, patient age, sex, and cognitive function assessed using the K-MMSE were included as covariates in the regression analyses.

### Statistical analysis

Descriptive statistics were used to summarize the demographic and clinical characteristics of the study participants. The normality of each variable was tested prior to correlation analysis. Depending on the distribution, either Pearson’s correlation coefficient (for normally distributed variables) or Spearman’s rank correlation coefficient (for non-normally distributed variables) was used to examine the relationships among FMA-L/L, BBS, and FAC scores on admission.

To examine the clinical utility of each assessment tool, binary logistic regression analyses were performed with independent ambulation at discharge (FAC ≥ 4) as the dependent variable. For the purpose of distinguishing concurrent association from predictive value, 2 sets of regression models were constructed:

(1) admission scores predicting admission FAC (association), and (2) admission scores predicting discharge FAC (prediction).

Separate models were created for FMA-L/L and BBS, and each model was adjusted for age, sex, and K-MMSE score.

Receiver operating characteristic (ROC) curve analyses were also conducted using the same 2 timepoint frameworks to assess the discriminative ability of FMA-L/L and BBS score. The area under the curve (AUC), optimal cut-off values, sensitivity, and specificity were calculated using Youden’s index (10). All statistical analyses were performed using IBM SPSS Statistics version 25.0 (IBM Corp, Armonk, NY, USA), and a *p*-value of less than 0.05 was considered statistically significant.

## RESULTS

Of the 211 patients initially screened, 109 met the eligibility criteria and were included in the final analysis. The mean age of participants was 64.51 ± 13.90 years, and 60.6% were male. The left side was affected in 56.9% of the patients. The average length of hospital stay was 25.94 ± 10.32 days, and the mean K-MMSE score on admission was 18.79 ± 8.84. On admission, the mean FMA-L/L was 22.43 ± 11.33, and the mean BBS score was 25.63 ± 20.47. Based on admission FAC scores, 22 patients (20.2%) were classified as independent ambulators (FAC ≥ 4), while 87 patients (79.8%) were non-independent. Based on discharge FAC scores, 32 patients (29.4%) were classified as independent in ambulation (FAC ≥ 4), while 77 (70.6%) remained non-independent. The general characteristics and initial and discharge functional assessments of participants are summarized in Table I.

**Table I:** General characteristics and initial functional assessments of participants (*n* = 109)

Variables	Mean ± SD
General characteristics	
Sex (male, %)	66 (60.6)
Affected side (left, %)	62 (56.9)
Age (years)	64.51 ± 13.90
Admission duration (days)	25.94 ± 10.32
Admission assessments	
K-MMSE	18.79 ± 8.84
FMA-L/L	22.43 ± 11.33
BBS	25.63 ± 20.47
FAC	Non-independent ambulators (FAC 0–3): 87 (79.8%) Independent ambulators (FAC 4–5): 22 (20.2%)
Discharge assessments	
K-MMSE	21.69 ± 8.37
FMA-L/L	26.17 ± 10.48
BBS	36.65 ± 19.90
FAC	Non-independent ambulators (FAC 0–3): 77 (70.6%) Independent ambulators (FAC 4–5): 32 (29.4%)

K-MMSE: Korean Mini-Metal State Examination; FMA-L/L: Fugl-Meyer Assessment for Lower Limb; BBS: Berg Balance Scale; FAC: Functional Ambulation Category.

Values are expressed as mean±standard deviation or percentage.

Discharge values for FMA-L/L, BBS, and K-MMSE are presented for descriptive purposes only and were not included in the regression or receiver operating characteristic analyses.

Spearman’s correlation analysis demonstrated significant positive associations among FMA-L/L, BBS, and FAC scores. The FMA-L/L score showed a strong correlation with BBS (*ρ* = 0.860, *p* < 0.01), and both were significantly correlated with discharge FAC scores. Specifically, the correlation coefficient between FMA-L/L and FAC was 0.546 (*p* < 0.01), and between BBS and FAC was 0.647 (*p* < 0.01). These findings indicate that higher motor and balance scores are moderately to strongly associated with greater levels of ambulatory independence on discharge. Detailed correlation coefficients are presented in Table II.

**Table II:** Spearman’s correlation between FMA-L/L, BBS, and initial functional assessments (*n*=109)

Item	FMA-L/L	BBS	FAC
FMA-L/L	1.000	0.860**	0.546**
BBS	0.860**	1.000	0.647**
FAC	0.546**	0.647**	1.000

FMA-L/L: Fugl-Meyer Assessment for Lower Limb; BBS: Berg Balance Scale; FAC: Functional Ambulation Category.

Values are expressed as Spearman’s ρ.

Binary logistic regression analyses were performed to evaluate the association and predictive value of admission FMA-L/L and BBS scores for independent ambulation (FAC ≥ 4).

In the association model (admission scores predicting admission FAC; Table III), both the FMA-L/L (Nagelkerke *R*² = 0.60) and BBS (*R*² = 0.75) models were statistically significant. FMA-L/L (*B* = 0.42, *p* < 0.001, Exp(*B*) = 1.52, 95% CI: 1.20–1.93) and BBS (*B* = 0.31, *p* < 0.001, Exp(*B*) = 1.37, 95% CI: 1.16–1.61) were significantly associated with independent ambulation. Among the covariates, sex was significant in the FMA-L/L model (*p* = 0.040), while age and K-MMSE were not significant in either model. In the prediction model (admission scores predicting discharge FAC; Table IV), both models were again significant (*R*² = 0.55 for FMA-L/L; 0.56 for BBS). FMA-L/L (*B* = 0.22, *p* < 0.001, Exp(*B*) = 1.24, 95% CI: 1.11–1.39) and BBS (*B* = 0.10, *p* < 0.001, Exp(*B*) = 1.10, 95% CI: 1.05–1.15) significantly predicted independent ambulation on discharge. Sex was also significant in the FMA-L/L model (*p* = 0.015), whereas age and K-MMSE were not significant in either model.

**Table III:** Logistic regression models for the association between admission FMA-L/L or BBS scores and independent ambulation on admission (FAC ≥ 4)(*n*=109)

Independent variable	Regression coefficient (*B*)	SE	*p*-value	Exp (*B*)	CI	Nagelkerke *R*²
Model for FMA-L/L						
FMA-L/L	0.42	0.12	< 0.001**	1.52	1.20–1.93	0.60
Age	–0.06	0.03	0.088	0.95	0.89–1.02	
Sex	–1.56	0.76	0.040*	0.21	0.05–0.93	
K-MMSE	0.03	0.05	0.514	1.03	0.94–1.14	
Intercept	–9.18	4.06	< 0.001**			
Model for BBS						
BBS	0.31	0.08	< 0.001**	1.37	1.16–1.61	0.75
Age	–0.01	0.04	0.821	0.99	0.92–1.07	
Sex	–1.26	0.99	0.200	0.28	0.04–1.95	
K-MMSE	–0.04	0.07	0.565	0.96	0.84–1.10	
Intercept	–11.60	4.79	< 0.001**			

Each model examined the association of admission clinical scores (FMA-L/L or BBS) and ambulation status on admission.

All models were adjusted for the following covariates: age, sex, and K-MMSE.

* *p* < 0.05,

** *p* < 0.01, statistically significant difference.

FAC: Functional Ambulation Category; FMA-L/L: Fugl-Meyer Assessment for Lower Limb; BBS: Berg Balance Scale; K-MMSE: Korean Mini-Metal State Examination; SE: standard error; CI: confidence interval.

**Table IV:** Logistic regression models predicting independent ambulation on discharge (Functional Ambulation Category ≥ 4) from admission FMA-L/L or BBS scores (*n* = 109)

Independent variable	Regression coefficient (*B*)	SE	*p*-value	Exp (*B*)	CI	Nagelkerke *R*²
Model for FMA-L/L						
FMA-L/L	0.22	0.06	< 0.001**	1.24	1.11–1.39	0.55
Age	–0.05	0.02	0.053	0.95	0.90–1.00	
Sex	–1.56	0.64	0.015*	0.21	0.06–0.74	
K-MMSE	0.04	0.04	0.291	1.05	0.96–1.13	
Intercept	–2.44	2.18	0.087			
Model for BBS						
BBS	0.10	0.02	< 0.001**	1.10	1.05–1.15	0.56
Age	–0.04	0.02	0.125	0.96	0.92–1.01	
Sex	–1.07	0.64	0.093	0.34	0.10–1.20	
K-MMSE	0.01	0.04	0.914	1.01	0.92–1.09	
Intercept	–0.38	2.01	< 0.001**			

All models were adjusted for the following covariates: age, sex, and K-MMSE.

Each model assessed whether admission clinical scores (FMA-L/L or BBS) predicted independent ambulation on discharge (FAC ≥ 4).

FMA-L/L: Fugl-Meyer Assessment for Lower Limb; BBS: Berg Balance Scale; K-MMSE: Korean Mini-Metal State Examination; SE: standard error; CI: confidence interval.

* *p* < 0.05,

** *p* < 0.01, statistically significant difference.

**Fig. 1:**
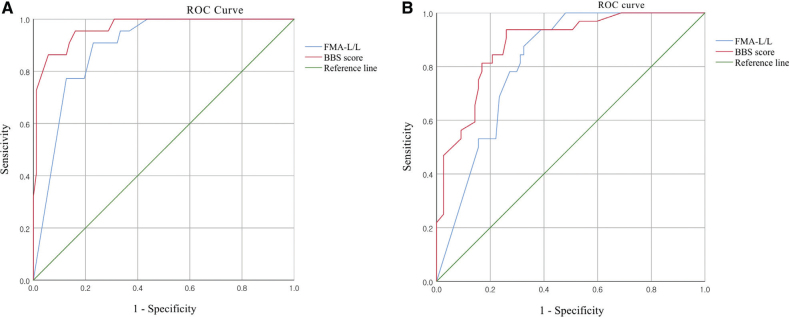
Receiver operating characteristic curves for classifying independent ambulation (functional ambulation category ≥ 4) using admission FMA-L/L and BBS scores. (A) Concurrent classification based on ambulation status on admission Functional Ambulation Category, (B) Predictive classification of ambulation status on discharge Functional Ambulation Category. ROC: Receiver operating characteristic; FMA-L/L: Fugl-Meyer Assessment for Lower Limb; BBS: Berg Balance Scale.

ROC curve analyses were conducted to evaluate the concurrent and predictive classification performance of admission FMA-L/L and BBS scores for identifying independent ambulation (FAC ≥ 4). For the concurrent classification model (admission scores predicting admission FAC), the area under the curve (AUC) was 0.89 (95% CI: 0.83–0.95, *p* < 0.001) for FMA-L/L and 0.97 (95% CI: 0.93–1.00, *p* < 0.001) for BBS. The optimal cut-off values determined using Youden’s index were 33.5 for FMA-L/L (sensitivity = 0.77, specificity = 0.87) and 47.5 for BBS (sensitivity = 0.86, specificity = 0.94). For the predictive classification model (admission scores predicting discharge FAC), the AUC was 0.82 (95% CI: 0.74–0.89, *p* < 0.001) for FMA-L/L and 0.88 (95% CI: 0.82–0.95, *p* < 0.001) for BBS. The optimal cut-off value was 29.5 for FMA-L/L (sensitivity = 0.78, specificity = 0.73) and 38.5 for BBS (sensitivity = 0.81, specificity = 0.83). Detailed results of the ROC analysis are presented in Table V, and the corresponding ROC curves are illustrated in Fig. 1.

**Table V:** ROC analysis for the concurrent and predictive classification of independent ambulation (FAC ≥ 4) using FMA-L/L and BBS scores (*n* = 109)

Variable	AUC	*p*-value	CI	Cut-off value	Sensitivity	Specificity
Admission (concurrent classification)						
FMA-L/L	0.89	< 0.001**	0.83–0.95	33.5	0.77	0.87
BBS	0.97	< 0.001**	0.93–1.00	47.5	0.86	0.94
Admission to discharge (predictive classification)						
FMA-L/L	0.82	< 0.001**	0.74–0.89	29.5	0.78	0.73
BBS	0.88	< 0.001**	0.82–0.95	38.5	0.81	0.83

Cut-off, sensitivity, and specificity were calculated using Youden’s index.

ROC: receiver operating characteristic; FAC: Functional Ambulation Category; AUC: area under the curve; FMA-L/L: Fugl-Meyer Assessment for Lower Limb; BBS: Berg Balance Scale.

** *p* < 0.01.

## DISCUSSION

This study demonstrated that admission scores on both the Fugl-Meyer Assessment for FMA-L/L and the BBS were significantly associated with independent ambulation (FAC ≥ 4) on both admission and discharge in patients with subacute stroke. These findings suggest that lower limb motor function and balance ability are both clinically meaningful indicators for assessing and predicting walking independence in the early stages of stroke rehabilitation.

ROC curve analyses confirmed the classification performance of both tools. The BBS showed excellent accuracy in identifying current ambulatory status, while the FMA-L/L demonstrated significant predictive value for discharge ambulation. Although the BBS yielded slightly higher AUC values, the FMA-L/L remains clinically important as it directly reflects motor capacity and control.

These findings are consistent with previous studies. Mao et al. (11) reported that the BBS was a strong predictor of gait function in subacute stroke patients, and Alzahrani et al. (12) found that BBS scores were highly correlated with walking independence. Similarly, the FMA-L/L has been widely used as a tool to evaluate lower limb recovery, and its relationship with gait outcomes has been well documented. Building on these findings, the present study shows that both assessments can be applied in the early rehabilitation phase to estimate not only current function but also the likelihood of future recovery.

Clinically, these simple bedside tools may help prioritize treatment decisions even for patients who are not yet ambulatory. Higher scores may justify the early initiation of intensive gait training, while lower scores may indicate the need to focus on fall prevention, caregiver education, or compensatory strategies. For patients in the early, non-ambulatory phase, this approach can support more efficient allocation of rehabilitation resources and better-informed planning. Several limitations should be considered. First, the retrospective single-centre design limits the generalizability of the findings. Second, although the regression models were adjusted for age, sex, and cognitive function, other important factors such as lower limb strength, spasticity, depression, fear of falling, and pre-stroke walking ability were not included. In particular, pre-stroke ambulation was assessed only through self-report, which may not reflect actual community-level independence.

In conclusion, the BBS and FMA-L/L are clinically useful tools for assessing and predicting independent ambulation in patients with subacute stroke. When used together on admission, they can support early rehabilitation planning and individualized treatment strategies, even for patients who are not yet able to walk.
